# Mixture toxicity of six pharmaceuticals towards *Aliivibrio fischeri*, *Daphnia magna*, and *Lemna minor*

**DOI:** 10.1007/s11356-021-17928-y

**Published:** 2021-12-14

**Authors:** Anna Białk-Bielińska, Łukasz Grabarczyk, Ewa Mulkiewicz, Alan Puckowski, Stefan Stolte, Piotr Stepnowski

**Affiliations:** 1 Department of Environmental Analysis, Faculty of Chemistry, University of Gdańsk, ul. Wita Stwosza 63, 80-308 Gdańsk, Poland; 2 Institute of Water Chemistry, Technische Universität Dresden, 01062 Dresden, Germany

**Keywords:** Emerging pollutants, Joint effects, Ecotoxicity, Aquatic environment, Toxicity test, Concentration addition, Independent action

## Abstract

As the knowledge on the joint effects of pharmaceuticals towards different non-target organisms is still limited, the aim of our study was to evaluate the toxicity of mixtures of pharmaceuticals, as well as their baseline toxicity towards three selected organisms, namely the bioluminescent bacteria *Aliivibrio fischeri*, the crustacean *Daphnia magna*, and the duckweed *Lemna minor.* Different mixtures composed of three up to five pharmaceuticals having the same or different mechanisms of action in terms of their therapeutic activity (non-steroidal anti-inflammatory drugs, opioid analgesic, antibacterial and anti-epileptic drugs) were investigated. The observed EC_50_s were compared with those predicted using the concentration addition (CA) and independent action (IA) models. In general, the EC_50_ values for mixtures predicted with the CA model were lower than those obtained with the IA model, although, in some cases, test predictions of these two models were almost identical. Most of the experimentally determined EC_50_ values for the specific mixtures were slightly higher than those predicted with the CA model; hence, a less than additive effect was noted. Based on the obtained results, it might be concluded that the CA model assumes the worst-case scenario and gives overall closer predictions; therefore, it should be recommended also for modeling the mixture toxicity of pharmaceuticals with different modes of action.

**Supplementary Information:**

The online version contains supplementary material available at 10.1007/s11356-021-17928-y.

## Introduction

During the last decades, the problem of the presence of residues of pharmaceuticals (recognized as emerging pollutants) in the environment has gained huge scientific attention (Ankley et al. 2007; Kümmerer, 2010; Rodriguez-Mozaz et al. 2010; aus der Beek et al. 2016; Klatte et al. 2017; Pereira et al. 2020; Vasilachi et al. 2021). Since the first detection of pharmaceuticals in environmental samples, numerous studies related to this issue have been published. These include the development of very selective and sensitive analytical procedures (Zrnčić et al. 2014), the application of different methods for their removal from a variety of environmental samples (Caban and Stepnowski 2021), and the evaluation of their fate and possible effects (Kümmerer, 2010; Brausch et al. 2012). Even though the state of knowledge on this topic has significantly improved, the potential threats posed by the presence of pharmaceuticals are still not sufficiently recognized.

Currently, the Environmental Risk Assessment (ERA) of pharmaceuticals is mainly carried out for individual substances (CHMP 2006; CVMP 2008; VICH 2000, 2005). However, they are present in the environment in different mixtures. For example, in the recently published report of German Environment Agency (Dusi et al. 2019) based on the data presented in 504 peer-reviewed articles published in 2010–2016, 771 pharmaceutical substances were detected in 75 countries worldwide. This proves how complex is the problem of the presence of the mixtures of pharmaceuticals in the environment. Godoy and Kummrov (2017) in the review paper that presents the summarized state of knowledge on the ecotoxicity of pharmaceuticals and personal care product mixtures based on the 194 assessments of the toxicity of mixtures from 65 articles published during 2000–2017. The authors also highlight future needs and indicate trends for performing such mixture studies.

Published ecotoxicological data prove that mixture effects of different pharmaceuticals towards non-target organisms may be higher than those predicted on the basis of the single components toxicity data (e.g. Cleuvers 2003; Eguchi et al. 2004; Drzymała and Kalka 2020). Although the results of studies on the mixture toxicity of pharmaceuticals are available (Table 1), new research on this subject may contribute to improve our understanding of the consequences of their presence in the environment. In Table 1, selected available literature data on the toxicity of mixtures of pharmaceuticals towards aquatic organisms representing three selected in our study tropic levels (bacteria, higher plants, or crustacean) mainly referring to standard endpoints in acute toxicity tests are presented.

**Table 1:** Selected literature data available on the mixture toxicity of pharmaceuticals

Mixture composition		Tested organisms	Conclusions	Reference
Non-specifically acting pharmaceuticals	Carbamazepine Diclofenac Fluoxetine Gemfibrozil Naproxen	Bioluminescent bacteria *Photobacterium leiognathi*	The applied mixture toxicity models (CA, IA, TSP) were all in agreement with the experimental results. The CA model is suitable to predict the effects of mixtures composed of both specifically and non-specifically acting chemicals towards bacteria	(Neale et al. 2017)
Specifically acting pharmaceuticals	Doxycycline Monensin Sulfamethiazole Sulfamethoxazole Tetracycline			
Specifically + non-specifically acting pharmaceuticals	Carbamazepine Diclofenac Fluoxetine Gemfibrozil Naproxen Doxycycline Monensin Sulfamethiazole Sulfamethoxazole Tetracycline			
Mixtures of 10 up to 56 organic micropollutants including pharmaceuticals		Bioluminescent bacteria *Aliivibrio fischeri*	The CA model sufficiently described the toxic effect of tested mixtures	(Tang et al. 2013)
Mixtures composed of 2 up to 12 chemicals belonging to the group of baseline toxicants (polar and non-polar chemicals) + ionisable chemicals (including pharmaceuticals)		Bioluminescent bacteria *Aliivibrio fischeri*	The CA model is suitable to predict the mixture effects of polar and non-polar chemicals	(Escher et al. 2017)
Sulpiride Clarithromycin Diphenhydramine HCl Bezafibrate Acetaminophen Ketoprofen Phenytoin Etodolac Crotamiton Epinastine HCl		Algae *Pseudokirchneriella subcapitata* Daphnid *Ceriodaphnia dubia* Fish *Danio rerio*	Predictions by CA and IA models were almost identical for algae. However, for the test with daphnid the models slightly underestimated the observed mixture toxicity. In the fish embryo test the observed toxicity fell between the toxicity predicted by CA and IA models	(Watanabe et al. 2016)
Ciprofloxacin 17α-ethinylestradiol 5-fluorouracil		Crustaceans *Daphnia magna* and *Artemia salina* Cyanobacteria *Cyanosarcina sp.* Algae *Desmodesmus quadricauda*, *Raphidocelis subcapitata* Plant *Lemna minor*	The predictions by IA model either underestimated or overestimated the effects It was proved that the exposure to tested mixtures of pharmaceuticals even at low concentrations, which individually cause no harm to organisms, may cause the adverse effects in the aquatic environment	(Affek et al. 2018)
Diclofenac Ibuprofen Naproxen Acetylsalicylic acid		Crustacean *Daphnia magna* Algae *Desmodesmus subspicatus*	Mixture toxicity could be well predicted by the CA concept	(Cleuvers 2004)
Metformin Bisoprolol Ranitidine Sotalol		Crustacean *Daphnia similis* Fish *Danio rerio*	Most of the binary mixture effects were between the toxicity predicted by CA and IA models	(Godoy et al. 2019)
Binary mixtures of: Azithromycin (AZM) Erythromycin (ERM) Carbamazepine (CBA) Oxytetracycline (OTC) Dexamethasone (DXM) Diclofenac (DCF)		Bioluminescent bacteria *Aliivibrio fischeri*	OTC–DCF, OTC–CBA and DCF-CBA indicated synergism with respect to additive behavior (CA model) For OTC–AZM, OTC–ERM, DCF–AZM, and DCF–ERM antagonistic behavior with respect to the CA model was observed All other binary mixtures indicated additive behavior In the case of the DCF–AZM, DCF–ERM, and OTC–AZM mixtures the applicability of the IA model as proof of the independent toxic action of the chemicals was confirmed	(Ukić et al. 2019)
Sulfadiazine (SDZ) Sulfaguanidine (SGD) Sulfamerazine (SMR) Sulfadimethoxine (SDM) Sulfadimidine (SDMD) (SMZ) Sulfaquinoxaline (SQO) Trimethoprim (TMP) Binary mixtures of SDMD + 6 compounds		Crustacean *Daphnia magna*	Antagonistic interaction: SDMD + SMR SDMD + SDM SDMD + SDZ SDMD + SGD Complex interaction (synergism additivity and antagonism): SDMD + SQO Simple additivity: SDMD + TMP	(De Liguoro et al. 2009)
Sulfaguanidine Sulfaquinoxaline		Crustacean *Daphnia magna* Algae *Pseudokirchneriella subcapitata*	Additive (antagonistic) interaction	(De Liguoro et al. 2010)
Sulfadimidine Sulfapyridine Sulfamethoxazole Sulfadiazine Sulfisoxazole Sulfamonomethoxine Sulfachloropyridazine Trimethoprim		Bioluminescent bacteria *Aliivibrio fischeri*	Antagonistic interaction between sulfonamides and trimethoprim in the acute toxicity test Synergistic interaction between sulfonamides and trimethoprim in the chronic test	(Zou et al. 2012)
Amoxicillin Chlortetracycline Sulfamethiazole Diclofenac Acetylsalycilic acid 10 combinations of binary mixtures of compounds mixed in a ratio corresponding to their individual IC_10_ Multi-component mixture of the five compounds mixed in a ratio corresponding to their individual value of PNEC		Bioluminescent bacteria *Aliivibrio fischeri*	The toxicity of the multi-component mixture was well predicted by the CA and IA models; deviations from the model predictions were found for almost all of the binary mixtures. The deviations from the CA and IA models were greater at lower concentrations, particularly when diclofenac and amoxicillin were present in the mixture. The application of the Combination Index method confirmed, for at least half of the binary combinations, the clear presence of synergistic deviations at the lowest tested concentrations, with a tendency towards antagonism at the higher ones	(Di Nica et al. 2017)
Binary mixtures of diclofenac and sulfamethoxazole		Bioluminescent bacteria *Aliivibrio fischeri* Crustacean *Daphnia magna* Plant *Lemna minor*	The mixture showed the highest toxicity towards *L. minor* and was classified as very toxic (high toxicity) to aquatic organisms Neither the CA nor the IA model suitably predicted the actual toxicity of the mixture. Only for IA model a good fit in the case of the toxicity test towards *A. fischeri* was obtained. For the other experiments, the actual toxicity was higher than that predicted, which might suggest the occurrence of more complex interactions in the mixture. The mixture toxicity index showed that the tested pharmaceuticals exhibited synergistic or partial additive effects which depended on test organism and test duration	(Drzymała and Kalka 2020)
Binary mixtures of: 17α-ethynylestradiol (EE2) Methotrexate (MTX) Diclofenac (DCF) Fluoxetine (FLX)		Plant *Lemna minor*	The CA model and toxic unit approach gave similar mixture toxicity predictions, with binary mixtures of MTX and FLX or MTX and EE2 exhibiting synergistic effects. In contrast, mixtures of DCF with FLX, EE2, or MTX mostly showed additive effects	(Markovic et al. 2021)
Binary, ternary, and quaternary mixtures of: Simvastatin Metformin Diazepam Omeprazole		Bioluminescent bacteria *Aliivibrio fischeri*	Hormesis phenomena, synergism, antagonism were observed while testing mixtures of these pharmaceuticals Mathematical (CA and IA) models which predict mixture toxicity, although important, they may fail to predict the real joint effects	(Jacob et al. 2020)

For all the abovementioned reasons, more ecotoxicological studies evaluating the joint effects of pharmaceuticals on non-target organisms are required. The need for a more realistic ERA of pharmaceuticals has been identified by other scientists (Schmitt et al. 2010; Tarazona et al. 2010; Backhaus 2016).

Taking into consideration the number of possible variations of mixtures present in the environment, estimating the effects of mixtures from individual substance data is generally accepted. The application of modeling to assess mixture toxicity is the most common and best-suited approach (Kortenkamp 2009). In general, two different models—CA, concentration addition and IA, independent action—have been proposed to predict the toxicity of mixtures of substances with similar and different modes of action, respectively (Kortenkamp et al. 2009) and have been described in detail in the “Materials and methods” section. However, it must also be highlighted that there is a third mixture assessment method, which includes interactions between chemicals in the mixture, and refers to all joint effects (such as synergism or antagonism) that deviate from the concept of additivity. Nevertheless, based on the information presented in the Bopp et al. (2015) report, interactions at environmentally relevant concentrations (which are usually quite low) are rare and, if observed, they show deviation from CA predictions that are in general relatively small.

The main aim of our study was to evaluate the toxicity of different mixtures of pharmaceuticals belonging to different therapeutic groups (Table 2), hence presenting different modes of action. The toxicity towards three selected organisms, namely, the bioluminescent bacteria *Aliivibrio fischeri* (formerly known as *Vibrio fischeri)*, the crustacean *Daphnia magna*, and the duckweed *Lemna minor* was evaluated*.* The selection of pharmaceuticals and tested species was based on our previously published results revealing their toxicity towards these organisms (Grabarczyk et al. 2020). Detailed justification of selection of these specific pharmaceuticals can be also found in that paper; however, it must be at least highlighted that they belong to the most frequently consumed and detected drugs in environmental samples. Therefore, we considered it crucial to also investigate their joint effects. Moreover, pharmaceuticals are designed to be biologically active; however, in non-target organisms, they may act in a specific or non-specific (as baseline toxicants) way. It is already known, for example, that estrogens (due to their specific mode of action) have a negative effect on fish (Kidd et al. 2007). The antidepressant fluoxetine, in turn, by affecting the biosynthetic pathways involved in the production of energy in algae, acts through a different (unpredicted) specific mode of toxic action (Neuwoehner et al. 2009; Escher et al. 2011). Therefore, in order to determine whether the selected pharmaceuticals act as baseline toxicants in the standard acute test, the toxic ratio values were calculated. The investigated mixtures were composed of pharmaceuticals belonging to the same therapeutic group as well as those with different modes of action in order to evaluate the applicability of the CA and IA models for the prediction of their joint toxic effects.

**Table 2:**
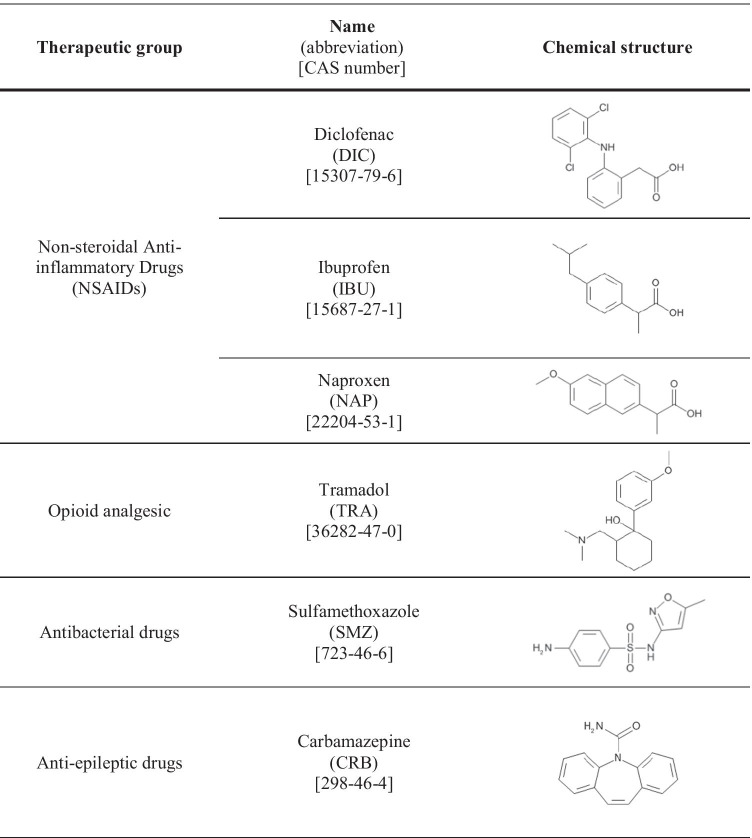
Investigated pharmaceuticals

## Materials and methods

### Chemicals

The pharmaceuticals and all reagents were purchased from Sigma-Aldrich (Steinheim, Germany). Pharmaceutical solutions were prepared immediately before the test in appropriate test medium. However, due to different water solubility, the stock solutions of CRB, SMZ, NAP, and IBU were prepared firstly in acetone at a concentration of 10 mg mL^−1^ and then diluted before the test in the proper test medium, so that the organic modifier content was not higher than 1% in the tested samples. On the contrary, DIC-sodium salt and TRA were always freshly prepared directly in the test medium due to their good solubility in water. Moreover, solvent controls were investigated in each test in order to verify that the addition of an organic solvent did not cause any effects.

### Investigated pharmaceutical mixtures

Nine different mixtures were tested with regard to the selected organisms (Table 3). The composition of each mixture was established based on the results obtained for single compounds, which were recently published by Grabarczyk et al. (2020). In general, in the mixture experiments, only pharmaceuticals with a determined EC_50_ values were investigated, as those with an EC_50_ assessed as higher than 100 mg L^−1^ according to the EC-Directive 93/67/EEC (European Commission 1993) are not considered to be harmful to aquatic organisms. Therefore, mixtures consisting of:

- DIC, IBU, NAP, and SMZ were tested towards *A. fischeri*;
- DIC, IBU, NAP, SMZ, and TRA were tested towards *D. magna*;
- DIC, IBU, NAP, SMZ, and CRB were tested towards *L. minor*.

**Table 3:** Single toxicity and the fraction of each chemical used in the different tested mixtures (mixtures were prepared by following an equi-toxicity concentration ratio design)

Organism	Mixture symbol	DIC	IBU	NAP	SMZ	CRB	TRA	*Σ*/EC_50_, CA
*A. fischeri*	**Single toxicity EC**_**50**_ **[mg L**^**-**^^**1**^**]**^*a*^	**11.62**	**14.97**	**25.17**	**51.77**	** > 100**	** > 100**	
	**M1 [mg L**^**-1**^**]**	3.83	4.94	8.31				17.08
	**M2 [mg L**^**-1**^**]**	2.91	3.74	6.29	12.94			25.88
*D. magna*	**Single toxicity EC**_**50**_ **[mg L**^**-1**^**]**^*a*^	**59.09**	**50.07**	**74.39**	**42.74**	** > 100**	**69.69**	
	**M1 [mg L**^**-1**^**]**	19.50	16.52	24.55				60.57
	**M2 [mg L**^**-1**^**]**	11.82	10.01	14.88	8.55		13.94	59.20
*L. minor*	**Single toxicity EC**_**50**_ **[mg L**^**-**^^**1**^**]**^*a*^	**16.52**	**13.25**	**20.70**	**3.07**	**50.17**	** > 100**	
	**M1 [mg L**^**-1**^**]**	5.45	4.37	6.83				16.65
	**M2 [mg L**^**-1**^**]**	3.30	2.65	4.14	0.61	10.03		20.73
	**M3 [mg L**^**-1**^**]**		4.37		1.01	16.56		21.94
	**M4 [mg L**^**-1**^**]**	4.13	3.31	5.17	0.77			13.38
	**M5 [mg L**^**-1**^**]**	4.13	3.31	5.17		12.54		25.15

^*a*^Data incorporated from our previous study (Grabarczyk et al. 2020)

Stock solutions of each mixture were prepared and the tests were performed in a series of dilutions to achieve full dose–response curves. The mixture concentrations ranged from 3.125 up to 100 mg L^−1^ for the tests with *L. minor*, from 1 up to 50 mg L^−1^ for the tests with *A. fischeri*, and from 20 up to 100 mg L^−1^ for the tests with *D. magna*.

It must be highlighted that the mixture studies were performed immediately after experiments evaluating the toxicity of single compounds in order to obtain reliable results. The fraction of the individual pharmaceutical in the specific mixture was determined based on the EC_50_ values for single pharmaceuticals and the CA model, which is presented in Table 3.

### Ecotoxicological tests

All the ecotoxicological tests were carried out according to the recommended OECD or ISO guidelines. In order to check the test procedure and validity of the test reference substances (3,5-dichlorophenol in the tests with *L. minor*, potassium dichromate in the tests with *A. fischeri* and *D. magna*) were tested at least twice during the test time period. Each of the tests was performed in two or more independent replicates. The exact number of replicates and concentrations tested within each test is described below. The dose–response curves and the EC_50_ values of the investigated mixtures were obtained using the drfit package in the R language and environment for statistical computing (http://www.r-project.org) (R Core Team, 2014). The mathematical formulas applied for this purpose are presented and described in the Supplementary Material.

#### Aliivibrio fischeri

The luminescence inhibition assay was performed according to the ISO11348-3:2007 guideline (2007) using the LCK 482 test kit (Dr. Lange GmbH, Germany). A detailed description of this test is presented in our previous paper (Grabarczyk et al. 2020). Briefly, the test was performed at 15 °C. The pH of the test medium and tested solutions was 7. The bioluminescence inhibition was measured after 30 min. Each test consisted of a control (including solvent controls) and eight different concentrations (1, 2.5, 5, 10, 20, 30, 40, and 50 mg L^−1^) of the investigated mixtures (M1 and M2) in two replicates.

#### Daphnia magna

The acute immobilization test with *D. magna* was carried out using DAPHTOXKIT F (MicroBioTest Incorporation, Gent, Belgium), which was performed based on the OECD 202 guideline (OECD 202, 2004). All the details of this test are presented in our previous paper (Garbarczyk et al. 2020). Briefly, the test was performed for 48 h at 20 °C (± 1 °C) in the darkness and the pH of the medium was kept within a range of 6 to 8. Each test consisted of a control (including solvent controls) and five different concentrations (20, 40, 60, 80, and 100 mg L^−1^) of the investigated mixtures (M1 and M2) in four replicates.

#### Lemna minor

The *L. minor* growth inhibition test (7 days) was performed in accordance with the OECD 221 guideline (OECD 221, 2006) and is described in detail in our previous paper (Grabarczyk et al. 2020). Briefly, the test was performed in a climate chamber at 25 °C (± 1 °C) under irradiation of 6000 lx and with a humidity of 60%. The pH value of the test medium and all tested solutions was 5.5 (± 0.5). Every test included six different concentrations (3.125, 6.25, 12.5, 25, 50, and 100 mg L^−1^) of each mixture tested (M1–M5) in three replicates, six controls, and six solvent controls.

### Applied models

The applied models for predicting the mixture toxicity, as well as to determine the baseline toxicity of investigated pharmaceuticals, are presented and described in Table 4. To calculate EC_50_ (IA), the experimental EC_50_ values for the single substances and the calculated percentage of the individual components in the mixture (Table 3) were used. Then, the concentrations of individual components of the mixture were determined so that the effects caused by them, calculated in the R program and substituted for the equation given in Table 4, would give the value *E*(*c*_mix_) equal to 0.5. The concentrations of individual components of the mixture and the effects caused by them calculated in the R program are presented in Table 4S.

**Table 4:** Models applied in our study

Purpose	Applied models	Comment	References
Mixture toxicity prediction	**• Concentration addition (CA)** $\sum \frac{{C}_{i}}{{EC}_{50i}}=1$ where: C_i_ – the concentration of component *i* in the mixture; EC_50i_ – the EC_50_ value of component *i* as a single compound	It assumes that each chemical in a mixture contributes, in proportion to its dose, to the toxicity of the whole mixture, expressed as the percentage of the dose of that chemical alone which would be needed to produce the given effect of the mixture. This is the default model for predicting the toxicity of the mixtures composed of chemicals with the same molecular mechanisms of action	(Cleuvers 2003; 2004)
	**• Independent action (IA)** $E\left({c}_{mix}\right)=E\left({c}_{1}+...+{c}_{n}\right)=1- \prod_{i=1}^{n}\left[1-E({c}_{i})\right]$ where: c_i_ – concentration of the i^th^ component; E(c_i_)—effect of component i if applied solely at that concentration at which it is present in the mixture; *E*(*c*_mix_)—the effect of the total mixture (scaled from 0–1) at concentration equal to the sum of c_i_	It considers mixtures that cause the same toxic effects. It accounts however for the impacts of toxic substances on various biological components. This model is useful to assess the toxicity of mixtures where the components pose a different modes of action	(Backhaus and Faust 2012; Cleuvers 2003)
Baseline toxicity assessment	${D}_{lipw}\left(pH\right)=\sum_{i=1}^{n}{\alpha }_{i} \times {K}_{lipw}(i)$		(Escher et al. 2020)
	$log{K}_{lipw}=0.90 log{K}_{ow}+0.52$		(Vaes et al. 1997)
	${D}_{lipw }\left(pH\right)= {\alpha }_{neutral} \times {K}_{lipw} \left(neutral species\right)+\left(1-{\alpha }_{neutral}\right) \times {K}_{lipw}(charged species)$ where: ${\alpha }_{\mathrm{neutral}}-$ fraction of the neutral species calculated using equitation below: for acids:$\alpha =\frac{1}{1+{10}^{pH-pKa}}$ for bases:$\alpha =\frac{1}{1+{10}^{pKa-pH}}$		(Escher et al. 2020)
	**•** QSAR model for predicting the baseline toxicity towards bacteria, *Aliivibrio fischeri* (30 min bioluminescence inhibition): $\mathrm{log}\left(\frac{1}{EC50\left[M\right]}\right)= 0.75 log{D}_{lipw}\left(pH 7\right)+ 0.97$ **•** QSAR model for predicting the baseline toxicity towards *Daphnia magna* (48 h – immobilization): $\mathrm{log}\left(\frac{1}{EC50\left[M\right]}\right)= 0.90\mathrm{log}{D}_{lipw }\left(pH 7\right)+ 1.61$		(Escher et al. 2017) (Escher et al. 2006)
	$TR= \frac{EC50 \mathrm{baseline}}{EC50 \mathrm{expr}}$	If *TR* is in the range from 0.1 up to 10 baseline toxicants, while those with a *TR* > 10 have a specific effect in the assay	(Verhaar et al. 1992)

The accuracy of the CA model predictions was verified by the application of the the model deviation ratio (MDR) approach (Belden et al. 2007; Markovic et al. 2021). This factor was calculated by dividing the predicted effective concentration (EC_50_) by the experimentally observed effective concentration of the mixture for the 50% effect. In order to determine if a pharmaceutical acts as baseline toxicant (act via non-specific effect) or had a specific effect in the whole organism, the experimental EC_50_ values obtained for individual compounds were compared with those predicted using QSARs models—available in the literature (Escher et al. 2006; 2017) for baseline toxicity. As for *L. minor*, such QSAR models are not available; this procedure was applied for *A. fischeri* and *D. magna.* It must be highlighted that most of the baseline QSAR models were developed for inert organic molecules and are based on the octanol–water partition coefficient (*K*_ow_) used as the descriptor of hydrophobicity. However, pharmaceuticals are in general ionizable compounds (classified as weak acids or bases) (Tarazona et al. 2010), and in their case, application of *K*_ow_ coefficient is unsuitable to measure bioaccumulation on biomembranes (recognized as the target site for non-specifically acting narcotic/baseline toxicants). For this purpose, the *K*_ow_ was replaced by the liposome-water distribution coefficient at a defined pH value (e.g. *D*_lipw_(pH 7)), which is determined using the equitation in Table 4 and is considered to be better descriptor (Escher et al. 2011). However, as hydrophobicity of a specific chemical, as well as its charge and interactions with the membrane, is crucial in partitioning into membranes, the *D*_lipw_(pH 7) parameter takes into account the speciation of organic acids and bases at pH 7 (Escher et al. 2020, 2011). If the experimental *D*_lipw_(pH 7) is not available in the literature, it can be calculated based on the value of the liposome-water partition coefficient of the neutral species (*K*_lipw_) and speciation based on the acidity constant (pKa). If *K*_lipw_ is not available, it can be calculated based on the *K*_ow_ value. For this purpose, we have gathered the literature data, and for missing information, we have performed proper calculations by applying the adequate equations and QSAR models, presented in the Table 4. In our study, we have applied the QSAR models for ionisable organic chemicals, which have been rescaled from the original QSAR models (based on log*K*_ow_) and are recommended by Escher et al. (2020). The determined EC_50_ values (based on these QSAR models) can be applied to identify whether specific compound (pharmaceutical) acts as a narcotic/baseline toxicant as well as to determine its specific toxicity level (expressed by the *TR* (toxic ratio) value—Table 4).

## Results and discussion

The determined experimental EC_50_ values for the investigated mixtures of pharmaceuticals, as well as their EC_50_ values predicted using the CA and IA models, are presented in Table 5. All obtained dose–response curves, as well as specific parameters describing dose–response curves, are presented in Figs. 1S–3S and in Tables 1S–3S in the Supplementary Material.

**Table 5:** Predicted vs. experimental EC_50_ [mg L^-1^] values of the investigated mixtures

Organism	Mixture	EC_50_ (CA)	EC_50_ (IA)	EC_50_ expr _(confidence interval)_	MDR	Effect*
*A. fischeri*	**M1** (DIC, IBU, NAP)	17.08	17.59	17.97 (17.35–18.63)	1.0	ad
	**M2** (DIC, IBU, NAP, SMZ)	25.88	31.87	35.58 (34.55–36.68)	0.7	ad
*D. magna*	**M1** (DIC, IBU, NAP)	60.57	135.52	67.14 (59.83–77.05)	0.9	ad
	**M2** (DIC, IBU, NAP, SMZ, TRA)	59.20	187.64	92.39 (84.31–107.33)	0.6	a
*L. minor*	**M1** (DIC, IBU, NAP)	16.65	29.62	10.35 (8.98–11.86)	1.6	s
	**M2** (DIC, IBU, NAP, SMZ, CRB)	20.73	30.77	21.39 (18.10–25.02)	1.0	ad
	**M3** (IBU, SMZ, CRB)	21.94	21.95	23.30 (20.81–26.06)	0.9	ad
	**M4** (DIC, IBU, NAP, SMZ)	13.38	20.40	15.89 (13.55–18.61)	0.8	ad
	**M5** (DIC, IBU, NAP, CRB)	25.15	40.39	24.00 (18.59–30.27)	1.0	ad

MDR model deviation ratio, * effect of the mixture evaluated based on MDR, ad - additive, a - antagonistic, s - synergistic

The investigated mixtures were composed of pharmaceuticals belonging to the same therapeutic group (e.g. M1 in each test) as well as those with different modes of action (M2 in the *A. fischeri* and *D. magna* tests and M2—M5 in the *L. minor* test) in order to evaluate the applicability of the CA and IA models for the prediction of their joint toxic effects.

However, in order to determine whether the selected pharmaceuticals act as baseline toxicants in the standard acute test, the *TR* values were calculated. In general, it is believed that baseline toxicity (narcosis) results from of the non-specific disturbance of the integrity and functioning of biological cell membranes by chemicals (mostly organic pollutants) by partitioning into these membranes (Escher et al. 2011). As these pollutants are not bound covalently to the membrane, this baseline toxicity is a reversible mechanism. Escher et al. (2011) highlighted that membrane disturbance is usually caused by the accumulation of specific pollutant in hydrophobic phases (like membrane lipids) within the organism; however, other mechanisms such as specific protein interactions cannot be excluded.

Based on the data presented in Table 6, it can be concluded that all pharmaceuticals acted specifically (*TR* > 10) in the test with *A. fischeri*, taking into account the new, quite recently rescaled QSAR model (Escher et al. 2017). However, different observations were made in the test with *D. magna*. The pharmaceuticals belonging to the group of non-steroidal anti-inflammatory drugs (NSAIDs), including DIC, IBU, and NAP, could be classified as baseline toxicants (determined *TR* values < 10). On the other hand, *TR* values exceeding 10 obtained for SMZ and TRA (60.2 and 22.3, respectively) indicated specific toxicity, which is in agreement with the results of the study of Escher et al. (2011). Moreover, the highest values of *TR* in the *A. fischeri* and *D. magna* tests proved that SMZ was the most specifically acting pharmaceutical, which was also observed by other authors who evaluated their toxicity in tests with other luminescence bacteria *Photobacterium leiognathi* (Neale et al. 2017) or green algae *Pseudokirchneriella subcapitata* (Escher et al. 2011).

**Table 6:** Selected properties of the investigated pharmaceuticals used to determine their baseline toxicity (*TR* value) in the tests

Compound	Molecular weight [g mol^-1^]	pKa	log*K*_ow_	log*K*_lipw_	pH-pKa	α_neutral_	α_neutral_ [%]	log*K*_lipw_ neutral	*K*_lipw_ (neutral species)	log*K*_lipw_ ionized	*K*_lipw_ (charged species)	*D*_lipw_ (pH 7)	log*D*_lipw_ (pH 7)	logEC_50_ baseline [M]	EC_50_ baseline [mg L^−1^]	EC_50_ experimental [mg L^-1^]	*TR*
*A. fischeri*																	
DIC	318.13	4.35	4.02	4.138	2.65	0.0022	0.22	4.45	28,183.83	2.64	436.52	498.50	2.70	− 2.993	323.12	11.62	27.8
IBU	206.29	4.45	3.79	3.931	2.55	0.0028	0.28	3.80	6309.57	1.81	64.57	82.12	1.91	− 2.406	810.32	14.97	54.1
NAP	230.26	4.36	3.1	3.31	2.64	0.0023	0.23	3.84	6918.31	1.92	83.18	98.80	1.99	− 2.466	787.33	25.17	31.3
SMZ	253.28	5.5	0.89	1.321	1.5	0.0307	3.07	1.32	20.89	0.32	2.09	2.67	0.43	− 1.289	13,008.98	51.77	251.3
CRB	236.27	13.9	2.45	2.725	− 6.9	1.0000	100.00	2.73	537.03	1.73	53.70	537.03	3.10	− 3.295	119.79	> 100	n.d
TRA	299.8	9.41	1.35	1.735	2.41	0.0039	0.39	1.735	54.33	0.735	5.43	5.62	0.75	− 1.532	8798.62	> 100	n.d
*D. magna*																	
DIC	318.13	4.35	4.02	4.138	2.65	0.0022	0.22	4.45	28,183.83	2.64	436.52	498.50	2.70	− 4.037	29.20	59.09	0.5
IBU	206.29	4.45	3.79	3.931	2.55	0.0028	0.28	3.80	6309.57	1.81	64.57	82.12	1.91	− 3.308	101.47	50.07	2.0
NAP	230.26	4.36	3.1	3.31	2.64	0.0023	0.23	3.84	6918.31	1.92	83.18	98.80	1.99	− 3.487	74.95	74.39	1.0
SMZ	253.28	5.5	0.89	1.321	1.5	0.0307	3.07	1.32	20.89	0.32	2.09	2.67	0.43	− 1.993	2571.84	42.74	60.2
CRB	236.27	13.9	2.45	2.725	− 6.9	1.0000	100.00	2.73	537.03	1.73	53.70	537.03	3.10	− 4.067	20.25	> 100	n.d
TRA	299.8	9.41	1.35	1.735	2.41	0.0039	0.39	1.735	54.33	0.735	5.43	5.62	0.75	− 2.285	1555.42	69.69	22.3

In general, it is commonly accepted that mixtures composed of chemicals with the same mode of action act according to the CA model. On the other hand, if all components exhibit a different modes of action, the IA model should be applied (Altenburger et al. 2003). However, for practical purposes, the CA model is usually recognized as a realistic worst-case scenario as the predicted mixture toxicity is usually within an order of magnitude of the experimental results (Altenburger et al. 2004). Based on the available literature data, most of the mixture studies with pharmaceuticals generally confirmed that the CA model adequately predicts the toxicity of the mixtures of pharmaceuticals from the same and different therapeutic groups (Table 1).

Hermens and Leeuwangh (1982) suggested that for multi-component mixtures consisting of chemicals posing different modes of action, where their individual concentrations are much below the individual toxic effect threshold, the underlying baseline toxicity may complement a substantial joint effect. Van Wezel and Opperhuizen (1995) stated that actually all chemicals exert a baseline toxicity, regardless of their specific mode of action. In complex mixtures with a defined toxicity, the number of constituents with different specific mechanisms of toxicity increases; however, the concentration of each chemical decreases. Therefore, their contribution to the overall toxicity by the non-specific baseline toxicity increases while that caused by the specific mode of toxic action decreases (Escher et al. 2011).

In most cases (except M1 in the *A. fischeri* test and M1 and M5 in the *L. minor* test), the experimentally determined EC_50_ values for the specific mixtures were slightly higher than those predicted with the CA model. Based on the obtained results, presented in Table 5, it can be noticed that the EC_50_ values predicted with the CA model were always lower than those obtained in the IA model. Therefore, it could be concluded that an additive or less than additive effect was noted. It was also confirmed by MDR analysis, which indicated additive toxicity (MDR value between 0.7 and 1.3) (Phyu et al. 2011) for most of the mixtures tested (Table 5). However, in some cases (mixture M1 and M2 in the *A. fischeri* test and M3 and M4 in the *L. minor* test), the CA and IA models gave very similar predictions (Table 5), which is quite common for mixtures of many compounds (Backhaus et al. 2000). This might result from the basic limitations of these two concepts, which may be correlated with the fact that they do not consider such factors as uptake, distribution, metabolism, and excretion of chemicals, which may have potential effects on the mixture toxicity.

The detected additive or less than additive interactions are in agreement with data from other authors (Brain et al. 2004; De Liguoro et al. 2009, 2010; Tang et al. 2013; Geiger et al. 2016; Escher et al. 2017; Neale et al. 2017). For example, Brain et al. (2004) observed that mixture toxicity of eight pharmaceuticals (belonging to different groups with different modes of action) to the aquatic macrophytes *Lemna gibba* and *Myriophyllum sibiricum* was additive. Moreover, De Liguoro et al. (2009, 2010) assessed that the toxicity of mixture of sulfamethazine with trimethoprim towards *D. magna*, as well as various binary mixtures of sulfonamides (sulfaquinoxaline and sulfaguanidine) towards *P. subcapitata* and *D. magna*, was less than additive.

On the other hand, Drzymała and Kalka (2020), who studied the toxicity of binary mixtures of two pharmaceuticals (diclofenac and sulfamethoxazole) belonging to different therapeutic groups, observed that none of the applied models (CA and IA) was suitable to predict the actual toxicity of the investigated mixtures. They have actually confirmed the interaction between mixture components, emphasizing that partial additive or even synergistic effects depended on the organisms tested and the duration of the test. Similarly, Markovic et al. (2021) observed that neither CA or IA models could predict the toxicity of the binary mixtures of methotrexate with 17α-ethynylestradiol or fluoxetine to *Lemna minor*, with both models underestimating the effect. The model deviation ratio calculated for these two mixtures was greater than 1.3, suggested synergistic effects. In turn, in the same study, the CA model was found to be the best fit for the toxicity of the binary mixtures of diclofenac and 17α-ethynylestradiol but slightly overestimated the toxicity of the mixtures of diclofenac with methotrexate or fluoxetine for which the predictions of the IA model were closer to the experimental results. However, MDR calculated for these two binary mixtures were greater than 0.7, suggesting that the CA model is still appropriate (Markovic et al. 2021). MDR analysis was also additionally applied in this study to verify the accuracy of the CA model predictions. Based on obtained results, it might be concluded that the CA model is still the most appropriate one. Only in one case (M1 in the *L. minor* test), MDR value was greater than 1.3 indicating synergistic effect (Table 5).

Even though the IA model is the recommended concept to predict the toxicity of chemicals with different modes of action, in our study, the mixture effects of pharmaceuticals with different modes of action were well predicted by CA model. Therefore, it seems to be sufficiently precautionary to use the CA model as a default approach with a relatively small probability of underestimating the toxicity, as it usually predicts higher toxicity than IA model.

The choice can be relevant in terms of assessing the risk posed by the residues of pharmaceuticals in the environment. If the CA model can be assumed, the risk quotient for the mixture can be calculated as the sum of risk quotients for individual pharmaceuticals (Escher et al. 2011).

However, Godoy and Kummrov (2017) in their review paper, concerning the mixture toxicity testing of pharmaceuticals and personal care products, highlight that this is still a very complex challenge and understanding the mechanisms and interactions involved in the joint action of these compounds are on special concern. Moreover, it is also highlighted that in the future, consistent criteria for prioritizing mixture components, selection of the test type in terms of their time duration, endpoint and level of biological organization, and selection of the adequate tools to predict and assess the obtained data should be established (Godoy and Kummrov 2017).

## Conclusions

A comparative report focused on the assessment of mixture toxicity of six pharmaceuticals belonging to different therapeutic groups towards the bacteria *A. fischeri*, the crustacean *D. magna*, and the duckweed *L. minor* has been presented for the first time. Mixture toxicity experiment was combined with the assessment of their mode of toxic action based on the application of appropriate QSAR models available for the test with *A. fischeri* and *D. magna* to determine whether the selected pharmaceuticals act as baseline toxicants in the standard acute test. Based on obtained results, it was concluded that all pharmaceuticals acted specifically (*TR* > 10) in the test with *A. fischeri*; however, different observations were made in the test with *D. magna*. The pharmaceuticals belonging to the group of NSAIDs (DIC, IBU, and NAP) could be classified as baseline toxicants. Despite these differences in their predicted mode of toxic action, the applied mixture toxicity models (CA and IA) were generally in good agreement with the experimental data. However, as the CA model in general assumes the worst-case scenario, and gives overall closer predictions, it can be recommended also for modeling the mixture toxicity of dissimilarly acting pharmaceuticals. The presented results contribute to a better understanding of the risks posed by the presence of these chemicals in the environment.

## Supplementary Information

Below is the link to the electronic supplementary material.

Supplementary file1 (DOCX 143 KB)

## Data Availability

Data associated with the present study can be accessed on request to the author (ewa.mulkiewicz@ug.edu.pl).
