# Supplementary material for: Mixture toxicity of six pharmaceuticals towards Aliivibrio fischeri, Daphnia magna, and Lemna minor
Source: Environ Sci Pollut Res Int. 2021 Dec 14;29(18):26977–91. doi: 10.1007/s11356-021-17928-y (PMC8989911; doi:10.1007/s11356-021-17928-y)
Supplement: Supplementary file 1 — Supplementary file1 (DOCX 143 KB) [file 11356_2021_17928_MOESM1_ESM.docx]

**Supplementary Material**

For

**Mixture toxicity of six pharmaceuticals towards *Aliivibrio fischeri*, *Daphnia magna* and *Lemna minor***

Anna Białk-Bielińska^1^, Łukasz Grabarczyk^1^, Ewa Mulkiewicz^1*^, Alan Puckowski^1^, Stefan Stolte^2^, Piotr Stepnowski^1^

^1^University of Gdańsk, Faculty of Chemistry, Department of Environmental Analysis, ul. Wita Stwosza 63, 80-308 Gdańsk, Poland

^2^Technische Universität Dresden, Institute of Water Chemistry, 01062 Dresden, Germany

*Corresponding author:

Ewa Mulkiewicz, ewa.mulkiewicz@ug.edu.pl, +48 58 523 51 90

**Mathematical forms of the model associated with concentration-response curves** (https://cran.r-project.org/web/packages/drfit/drfit.pdf)

The logistic model is widely used for sigmoidal dose response curves. For logistic model the four-parameter logistic function given by the formula (1) is used:

$$f\left( x,\left( b,c,d,e \right) \right)=c+\frac{d-c}{1+exp\{b\left( \log\left( x \right)-\log\left( e \right) \right)\}} (1)$$

with four parameters *b, c, d, e*. The parameter *e* is also denoted EC_50_ and it is the dose producing the response half-way between the upper limit, *d*, and lower limit, *c*. The parameter *b* denotes the relative slope around *e*.

When the lower limit is fixed at 0 (c=0) and the upper limit is fixed at 1 (d=1) the two-parameter logistic function is obtained and the equation (1) has the form:

$$f\left( x,\left( b,e \right) \right)=\frac{1}{1+exp\{b\left( \log\left( x \right)-\log\left( e \right) \right)\}} (2)$$

The equation (2) is used in logit fit.

**The description of parameters obtained for the dose-response curves**:

**sigma** - the square root of the estimated variance of the random error

**a** - this is the EC_50_ for the logit model.

**b** - this is the parameter b from equation (1) and (2).


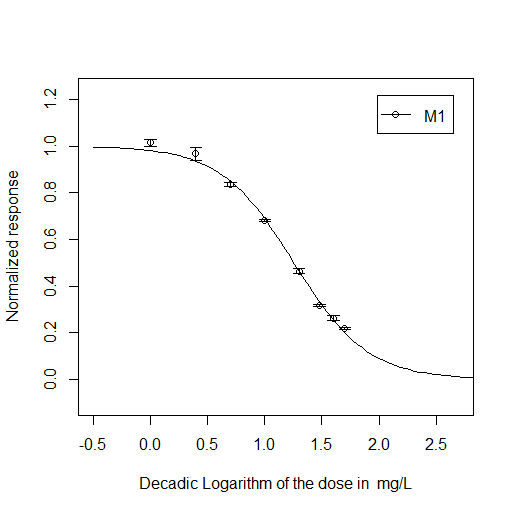

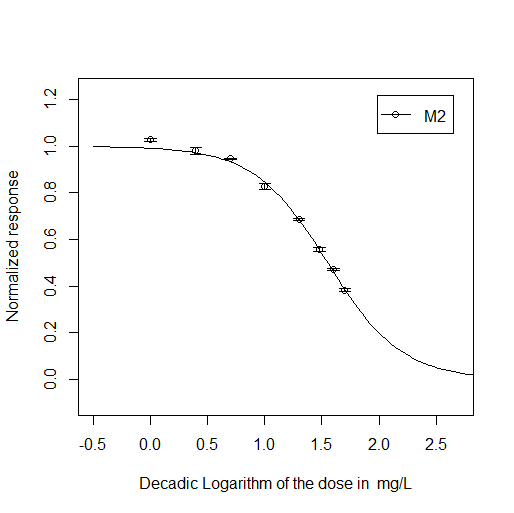


**Fig. 1S.** Dose-response curves obtained for studied mixtures of pharmaceuticals in *A. fischeri* luminescence inhibition assay

**Table 1S.** Parameters describing the dose-response curves obtained in *A. fischeri* luminescence inhibition assay

| **Mixture** | **M1** | **M2** |
| --- | --- | --- |
| **Model type** | logit | logit |
| **log*EC_50_*** | 1.255 | 1.551 |
| **2.5%** | 1.239 | 1.538 |
| **97.5%** | 1.270 | 1.564 |
| **sigma** | 0.023 | 0.019 |
| **a** | 1.255 | 1.551 |
| **b** | 0.321 | 0.323 |


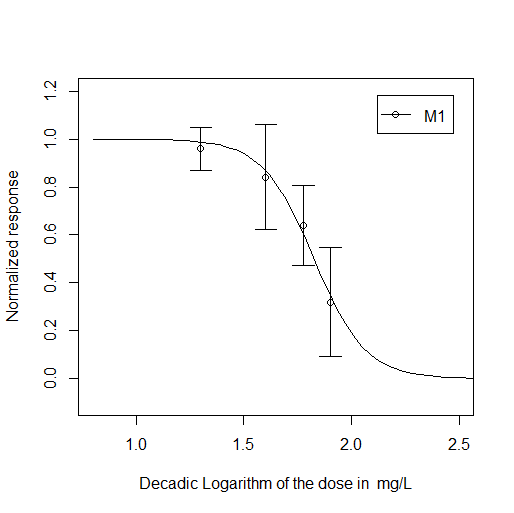

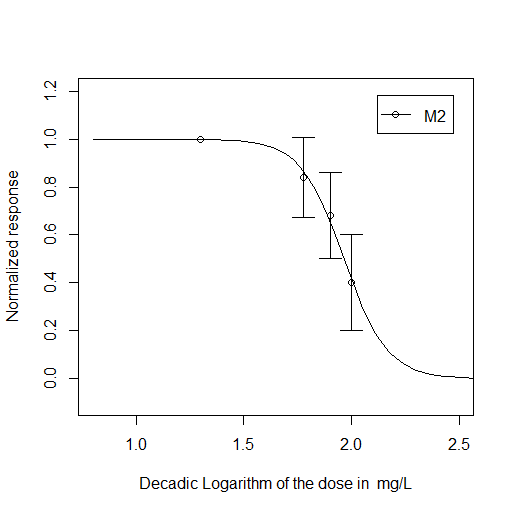


**Fig. 2S.** Dose-response curves obtained for studied mixtures of pharmaceuticals in *D. magna* acute immobilization test

**Table 2S.** Parameters describing the dose-response curves obtained in *D. magna* acute immobilization test

| **Mixture** | **M1** | **M2** |
| --- | --- | --- |
| **Model type** | logit | logit |
| **log*EC_50_*** | 1.827 | 1.966 |
| **2.5%** | 1.777 | 1.926 |
| **97.5%** | 1.887 | 2.031 |
| **sigma** | 0.156 | 0.151 |
| **a** | 1.827 | 1.966 |
| **b** | 0.119 | 0.101 |


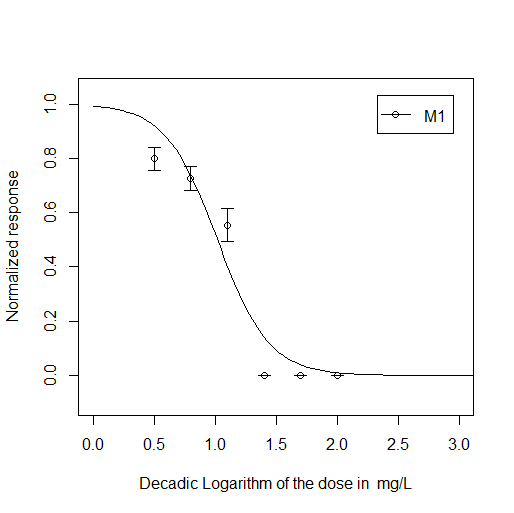

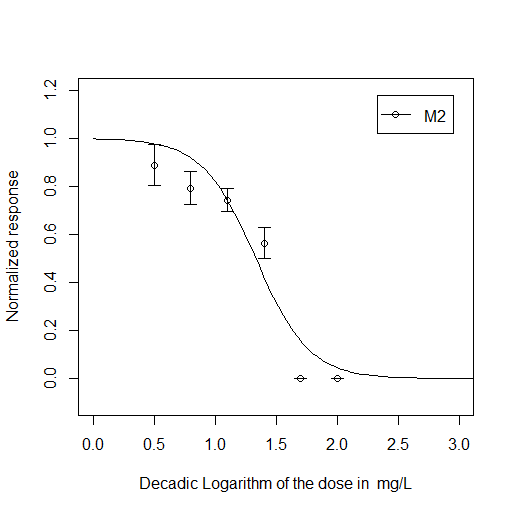

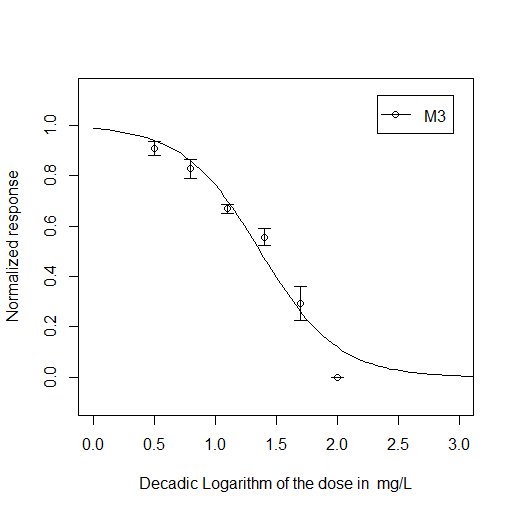

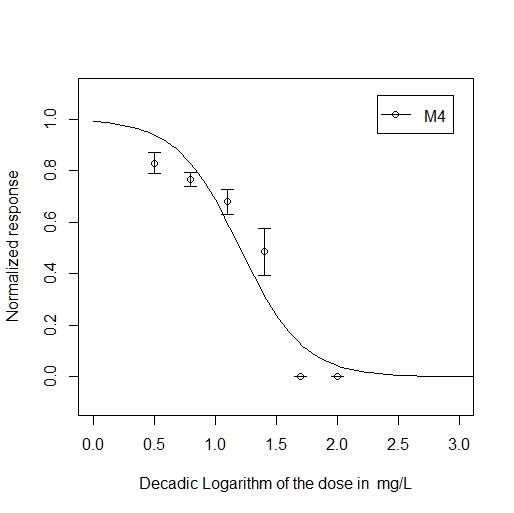

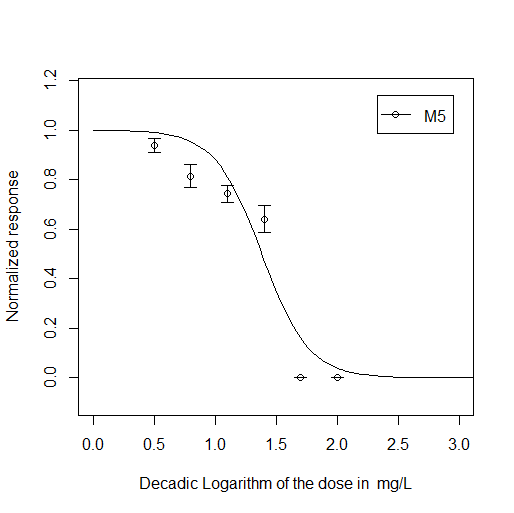


**Fig. 3S.** Dose-response curves obtained for studied mixtures of pharmaceuticals in *L. minnor* growth inhibition test

**Table 3S**. Parameters describing the dose-response curves obtained in *L. minnor* growth inhibition test

| **Mixture** | **M1** | **M2** | **M3** | **M4** | **M5** |
| --- | --- | --- | --- | --- | --- |
| **Model type** | logit | logit | logit | logit | logit |
| **log*EC_50_*** | 1.015 | 1.330 | 1.367 | 1.201 | 1.380 |
| **2.5%** | 0.953 | 1.258 | 1.318 | 1.132 | 1.269 |
| **97.5%** | 1.074 | 1.398 | 1.416 | 1.270 | 1.481 |
| **sigma** | 0.105 | 0.123 | 0.075 | 0.105 | 0.127 |
| **a** | 1.015 | 1.330 | 1.367 | 1.201 | 1.380 |
| **b** | 0.212 | 0.219 | 0.318 | 0.258 | 0.193 |

**Table 4S.** The concentrations of individual components of the mixture and the effects caused by them calculated in the R program used for the calculation of predicted EC_50_ (IA).

| Organism | Mixture | DIC | IBU | NAP | SMZ | CRB | TRA | Σ/  EC_50_, IA |
| --- | --- | --- | --- | --- | --- | --- | --- | --- |
| *A. fischeri* | **M1 [%]**  **[mg L^-1^]** | 22%  3.87 | 29%  5.10 | 49%  8.62 |  |  |  | 100%  17.59 |
|  | **Calculated effect** | 0.142 | 0.283 | 0.185 |  |  |  |  |
|  | **M2 [%]**  **[mg L^-1^]** | 11%  3.50 | 15%  4.78 | 24%  7.65 | 50%  15.94 |  |  | 100%  31.87 |
|  | **Calculated effect** | 0.124 | 0.272 | 0.162 | 0.067 |  |  |  |
| *D. magna* | **M1 [%]**  **[mg L^-1^]**  **Calculated effect** | 32%  43.37  0.179 | 27%  36.59  0.216 | 41%  55.56  0.226 |  |  |  | 100%  135.52 |
|  | **M2 [%]**  **[mg L^-1^]**  **Calculated effect** | 20%  37.53  0.093 | 17%  31.90  0.174 | 25%  46.91  0.156 | 14%  26.27  0.149 |  | 24%  45.03  0.073 | 100%  187.64 |
| *L. minor* | **M1 [%]**  **[mg L^-1^]**  **Calculated effect** | 33%  9.78  -0.072 | 26%  7.70  0.342 | 41%  12.14  0.292 |  |  |  | 100%  29.62 |
|  | **M2 [%]**  **[mg L^-1^]**  **Calculated effect** | 16%  4.92  -0.059 | 13%  4.00  0.202 | 20%  6.15  0.140 | 3%  0.93  0.176 | 48%  14.77  0.166 |  | 100%  30.77 |
|  | **M3 [%]**  **[mg L^-1^]**  **Calculated effect** |  | 20%  4.39  0.219 |  | 5%  1.1  0.218 | 75%  16.46  0.183 |  | 100%  21.95 |
|  | **M4 [%]**  **[mg L^-1^]**  **Calculated effect** | 31%  6.33  -0.074 | 25%  5.10  0.248 | 38%  7.75  0.180 | 6%  1.22  0.246 |  |  | 100%  20.40 |
|  | **M5 [%]**  **[mg L^-1^]**  **Calculated effect** | 16%  6.46  -0.075 | 13%  5.25  0.253 | 21%  8.48  0.199 |  | 50%  20.20  0.221 |  | 100%  40.39 |
